# Alcohol Intake and Abnormal Expression of Brf1 in Breast Cancer

**DOI:** 10.1155/2019/4818106

**Published:** 2019-10-31

**Authors:** Chenghao Huang, Yanmei Zhang, Shuping Zhong

**Affiliations:** ^1^State Key Laboratory of Molecular Vaccinology and Molecular Diagnostics, National Institute of Diagnostics and Vaccine Development in Infectious Diseases, School of Public Health, Xiamen University, China; ^2^Department of Pharmacology of Shantou University Medical College, China; ^3^Department of Medicine, Keck School of Medicine, University of Southern California, Los Angeles, CA, USA

## Abstract

Breast cancer is the most common malignant disease of females. Overall, one woman in every nine will get breast cancer at some time in her life. Epidemiological studies have indicated that alcohol consumption has most consistently been associated with breast cancer risk. However, the mechanism of alcohol-associated breast cancer remains to be addressed. Little is known about the effects of alcohol consumption on Brf1 (TFIIIB-related factor 1) expression and RNA Pol III gene (RNA polymerase III-dependent gene) transcription, which are responsible for protein synthesis and tightly linked to cell proliferation, cell transformation, and tumor development. Emerging evidences have indicated that alcohol induces deregulation of Brf1 and Pol III genes to cause the alterations of cell phenotypes and tumor formation. In this paper, we summarize the progresses regarding alcohol-caused increase in the expression of Brf1 and Pol III genes and analysis of its molecular mechanism of breast cancer. As the earlier and accurate diagnosis approach of breast cancer is not available yet, exploring the molecular mechanism and identifying the biomarker of alcohol-associated breast cancer are especially important. Recent studies have demonstrated that Brf1 is overexpressed in most ER+ (estrogen receptor positive) cases of breast cancer and the change in cellular levels of Brf1 reflects the therapeutic efficacy and prognosis of this disease. It suggests that Brf1 may be a potential diagnosis biomarker and a therapeutic target of alcohol-associated breast cancer.

## 1. Introduction

Alcohol intake is consistently associated with an increased risk of breast cancer [1–4]. Alcohol is known to promote mammary tumorigenesis [5–7]. Alcohol consumption is associated with ER+ (estrogen receptor positive) cases of breast cancer much more than with ER- cases of this disease [8–10]. Although the exact mechanism by which alcohol causes breast cancer is still unclear, alcohol consumption is thought to induce breast carcinogenesis through various mechanisms, such as the perturbation of estrogen metabolism and response, mutagenesis by the ethanol metabolite acetaldehyde, and oxidative damage, as well as by affecting the one-carbon metabolic pathways through reduced folic acid intake and use [11]. Previous studies have shown that chronic alcohol consumption results in the production of acetaldehyde and induction of CYP2E1. Acetaldehyde is a by-product of alcohol metabolism catalyzed by ADH (alcohol dehydrogenase), which has direct mutagenic and carcinogenic effects *in vitro* and *in vivo* studies [12]. CYP2E1 is associated with the release of ROS (reactive oxygen species) and conversion of procarcinogens to carcinogens. Alcohol exposure increases cellular production of ROS and induces cellular stress, resulting in tissue injury and ALD (alcoholic liver disease).

However, little is known about the role of alcohol in Brf1 (TFIIIB-related factor 1) expression and Pol III gene (RNA polymerase III-dependent gene) transcription during breast tumor development. Pol III genes are responsible for protein synthesis and tightly linked to cell proliferation, cell transformation, and cancer development. RNA polymerase III is responsible for the synthesis of a variety of small untranslated RNAs, including tRNAs and 5S rRNA [12]. Brf1 is a subunit of the TFIIIB complex of Pol III gene transcription machinery. Brf1 specifically regulates tRNAs and 5S rRNA transcription. The PNCs (perinucleolar compartments) are dependent on the continuation of RNA Pol III gene transcription and more prevalent in cancer cells than in normal cells [13]. PNC prevalence significantly correlates with the progression of breast cancer [14]. Accumulation of Pol III gene transcripts around the nucleolus is especially evident in transformed cells [15]. Consistent with the idea that a high translational capacity is necessary for rapid growth and proliferation of tumor cells, Pol III gene transcripts have been found to be increased in human cancers [10, 16, 17]. Furthermore, expression of the Pol III gene, BC200, was elevated in breast squamous cell carcinoma tissues [18]. Our studies using both cell culture models and animal models have revealed that alcohol induces transcription of tRNA^Leu^ and 5S rRNA [19, 20]. This induction in mice fed with alcohol is associated with tumor development [19]. Alcohol-induced tRNA and 5S rRNA transcription in a breast cancer cell line is in an ER*α*-dependent manner [20]. These evidences support the idea that ER*α* may mediate the modulation of alcohol-induced Brf1 expression and Pol III gene transcription in breast cells.

**Figure 1:**
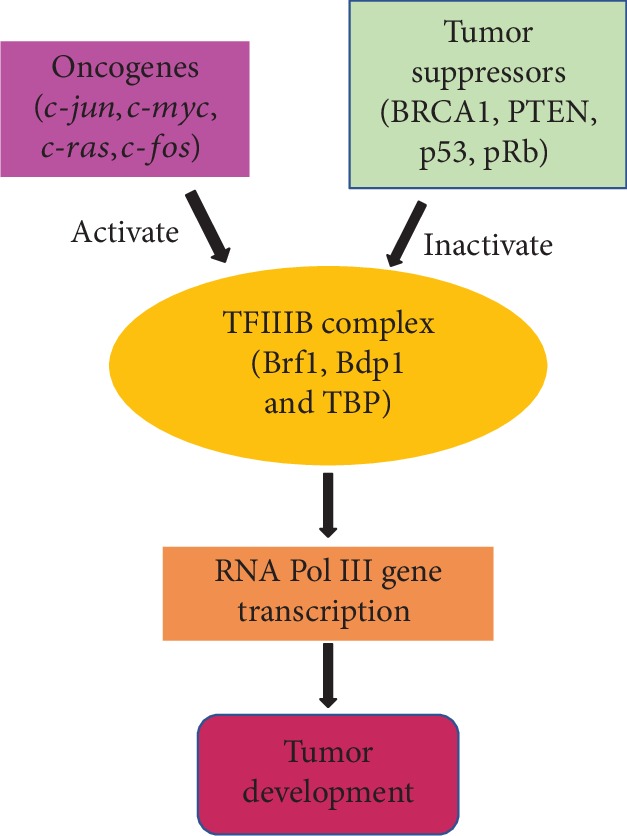
The alteration of the TFIIIB complex of transcription machinery. TFIIIB is a complex of the transcription machinery of RNA Pol III genes; TFIIIB includes Brf1, Bdp1, and TBP. Oncogenic proteins, such as c-Jun, c-Myc, Ras, and c-Fos activate TFIIIB to enhance RNA Pol III gene transcription, resulting in tumorigenesis. In contrast, tumor suppressors, such as BRCA1, PTEN, p53, and pRb, inactivate its activity to decrease the transcription of Pol III genes, leading to repression of tumor development.

Genome-wide studies have demonstrated that many RNA Pol II transcription factors are associated with RNA Pol III transcribed genes and overlap the occupancy of RNA Pol III [21]. Initial studies suggest that tRNAs are differentially expressed in a tissue-specific manner and that induction of RNA Pol III-dependent transcription may not uniformly increase all tRNA isoacceptors [22]. It implies that the differences of Pol III gene transcription levels match the diversities in various physiological and pathological conditions. The transcription machinery of tRNA genes is composed of RNA Pol III, TFIIIB, and TFIIIC complexes to control tRNA transcription, whereas, the 5S rRNA gene transcription machinery consists with the complexes of RNA Pol III, TFIIIB, TFIIIC, and TFIIIA. The complex of TFIIIB consists of TBP and its associated factors, Brf1 (or Brf2) and Bdp1. Brf1 and Bdp1 are specifically utilized in the Pol III gene transcription process. TBP is required for transcription by all three nuclear polymerases, Pol I, II, and III, in gene transcription [23]. The tumor suppressors, BRCA1, p53, pRb, and PTEN, repress, whereas oncogenes, such as Ras, c-Jun, c-Myc, and c-Fos, increase Pol III gene transcription [24–26] (Figure 1). An increase in TBP levels to induce Pol III gene transcription is required for its transforming function [27–29]. Studies have demonstrated that change in TBP expression is able to alter the cellular level of Bdp1, but not Brf1 [30]. It suggests that Brf1 is more specific and critically important in the regulation of tRNA and 5S rRNA genes. A recent study indicates that alcohol-induced TBP transcription is not significantly associated with ER*α* expression in ER+ breast cancer cells [20]. In contrast, alteration of alcohol-induced ER*α* expression significantly changes cellular levels of Brf1 in these cells [20]. This implies that this TFIIIB subunit, Brf1, may be involved in the ER-mediated induction of Pol III genes caused by alcohol. In the review, we will mainly focus on the role of alcohol-induced changes in Brf1 expression and deregulation of Pol III genes.

## 2. Alcohol-Caused Alteration of Brf1 Expression and Breast Cancer

In terms of the difference in regulation regions of RNA Pol III genes, these genes are divided into three types. Type I of Pol III genes is 5S rRNAs, and Type II of the genes is tRNAs, whereas Type III is composed of other small Pol III genes except type I and type 2 of these genes. Brf1 and Brf2 (TFIIB-related factor 2) are subunits of TFIIIB. Brf1 takes part in the regulation of Type 1 (5S rRNAs) and Type 2 (tRNAs) Pol III gene transcription, whereas Brf2 mainly modulates the transcription of Type III genes, such as U6, 7SL, and 7SK genes, and other small RNAs. As 5S rRNA and tRNAs function protein synthesis, they are required to cell growth, cell transformation, and tumor development. Brf1 gene IDs of human, mouse, and rat are 2972, 72308, and 299347, respectively. Human Brf1 gene (ID: 2972) primarily codes a 90 kD protein. At the review, we primarily focus on the discussions of abnormal Brf1 expression in the cases of human breast cancer and alcohol-induced Brf1 expression in breast cancer cells.

### 2.1. Abnormal Expression of Brf1 and Its Significance in Breast Cancer

As mentioned above, Brf1 specifically regulates transcription of tRNAs and 5S rRNA genes, while the deregulation of the Pol III genes is tightly linked to cell transformation and tumor development. This suggests that Brf1 plays a critically important role in breast cancer. However, the status of Brf1 expression in patients of breast cancer has been determined until recently. The studies report that, for the first time, 218 samples of human breast cancer have been collected to measure the levels of Brf1 expression by immunohistochemistry method. 102 of 218 cases (46.8%) display strong Brf1 staining in lesion tissues, which is classified in the high Brf1 expression group [10]. The other 116 cases (53.2%) of breast carcinoma include moderate, weak, or negative staining of Brf1 in the lesion tissues, which is classified as the low Brf1 expression group [10]. The staining signals of Brf1 in most of the breast cancer cases primarily accumulate in the nucleus, while its localization in the cytoplasm is only 3.7% (8/218) [10]. The most clinically pathological characteristic of 218 cases is not the significant correlation between Brf1 expression and other clinic pathological features, such as patient age, pausimenia, histological type, clinical stage, tumor size, lymph node, and metastasis [10]. In contrast, there is a significant correlation between high Brf1 expression and high ER expression (*p* = 0.012), high PR expression (*p* = 0.035) or non-triple-negative status (*p* = 0.012), but not Her2 expression (*p* = 0.357) [10]. These studies reveal that the levels of Brf1 expression of the human breast cancer are associated with their hormone statuses. The results are consistent with an early study, which demonstrates that ER*α* mediates alcohol-induced Brf1 expression [20]. More interestingly, the OS (overall survival) times of the patients with low Brf1 expression (118.7 ± 5.4 months, *n* = 116) are significantly shorter than with the cases of high Brf1 expression (137.5 ± 4.4 months, *n* = 102, *p* = 0.004) [10]. Furthermore, the DFS (disease-free survival) periods in the high Brf1 expression group (135.3 ± 5.0 months) are markedly longer than those in the low Brf1 expression group (112.8 ± 6.4 months, *p* = 0.004) [10]. Therefore, these studies show that patients with high Brf1 expression have better prognosis after hormone therapy. It looks strange, as high Brf1 expression in cancer patients should possess short survival times usually [17].

Approximately 80% cases of breast cancer are ER+ [4, 5, 31]. The ER+ patients were treated by hormone therapy after they were diagnosed as breast cancer and performed surgery resection, whereas a preferred drug of hormone therapy is Tam (tamoxifen), which is an antagonist of the estrogen receptor in breast tissue and has been widely used for the treatment for both early and advanced ER+ breast cancers in women [32]. We have demonstrated that high expression of Brf1 in hepatocellular carcinoma cases displays short OS period [17]. We also found the trend that the patients with high Brf1 expression reveal short OS period in other cancers (unpublished). More interestingly, our early studies have found that Tam significantly inhibits the induction of Brf1 caused by alcohol in ER+ breast cancer cells [33]. As Tam is able to repress Brf1 expression, the cellular levels of Brf1 in the ER+ patients are actually at low levels during hormone treatment. This explains why high Brf1 expression has longer survival periods in the ER+ cases than ER- cases with the disease. It suggests that cellular levels of Brf1 are not only as a diagnosis marker but also as an indicator of prognosis under hormone treatment.

### 2.2. Alcohol Increases Brf1 Expression to Promote Breast Tumor Development

Emerging evinces indicate that alcohol consumption is an established risk factor for breast cancer [1–4]. The relative increase in risk ranges from 5 to 10% (~1 drink/10 grams/day) to 40-50% (~3 drinks/day) [34, 35]. Studies have demonstrated that alcohol feeding prompted mammary tumor formation in mice [36, 37]. Mechanism analysis indicates that alcohol-promoted breast cancer development may be through its effect on the expression of aromatase and PPAR-*γ* with resulting increases in estrogen (ER ligand) levels [38, 39]. However, Brf1 overexpression in human cases of breast cancer is a direct evidence [10]. Mechanism studies demonstrate that alcohol increases Brf1 expression in ER+ breast cancer cells [20]. Alcohol induces transformation of nontumor breast cells, and it also increases the rates of colony formation of human breast cancer cells [20]. However, once knocking down Brf1 expression by its siRNA, the rates of colony formation will be significantly reduced. This indicates that alcohol-increased Brf1 expression is critically important in cell transformation and tumor development.

### 2.3. ER*α* and Runx2 Mediate Alcohol-Induced Brf1 Expression

Studies have demonstrated that alcohol increases the activity of the ERE-Luc promoter in ER+ MCF-7 cells [20] and alcohol intake enhances the transcriptional activity of ER*α* and activates the E2 signaling pathway (37.40). Alcohol increases cellular levels of Brf1 in ER+ breast cancer cells [20]. This implies that ER*α* has potential to mediate Brf1 expression. Studies support the idea that E2, a ligand of ER*α*, augments cellular levels of Brf1 mRNA and proteins. In contrast, ER*α* siRNA significantly represses Brf1 expression in ER+ breast cancer cells [20]. The capacity of ER*α* in Brf1 expression is not for another subunit, TBP, of TFIIIB complex.

Runx2 (Runt-related transcription factor 2) is a component of ER*α* downstream and is modulated by ER*α*. Runx2 is expressed in ER+ human breast cancer cell lines and participates in mammary gland development. Studies have indicated that Runx2 plays important roles in tumor cell growth and migration, as wells as in bone metastasis of breast cancer. However, it remains to be investigated whether Runx2 mediates Brf1 expression. Recently, we have determined the role of Runx2 in Brf1 expression. The results indicate that Runx2 is overexpressed in human biopsies of breast cancer (unpublished). Alcohol enhances Runx2 expression in ER+ breast cancer cell line, MCF-7. Repression of Runx2 decreases Brf1 expression (unpublished). ER*α* mediates alcohol-induced Runx2 expression in the cells (unpublished). Together, these studies demonstrate that Runx2 modulates Brf1 expression through the ER*α* pathway induced by alcohol. Alcohol-increased Runx2 expression may play a crucial role in alcohol-associated breast cancer development.

### 2.4. Alcohol Induces Signal Events Which Mediate Brf1 Expression

Brf1 is a phosphorylated protein. Plk1 (polo-like kinase 1) phosphorylates Brf1 on serine 450 to directly promote tRNA and 5S rRNA transcription in cell interphase [40]. This stimulatory modification is overridden at mitosis, when elevated Plk1 activity causes Brf1 phosphorylation on threonine 270, which prevents RNA polymerase III recruitment to the transcription machinery [40]. Studies have indicated that PKB (protein kinase B) phosphorylates Brf1 at serine 92, resulting in binding to 14-3-3 and impairment of mRNA decay activity [41]. Further analysis reveals that an additional regulatory site of Brf1 is at serine 203, which cooperates with its serine 92 *in vivo* [42]. Brf1 serine 203 is also phosphorylated by PKB. Mutation of both serine 92 and serine 203 to alanine uncouples Brf1 from PKB regulation, leading to constitutive mRNA decay even in the presence of stabilizing signals [42]. The deregulation of Pol III gene transcription has been linked to cancer, whereas germline mutations of genes encoding RNA polymerase III subunits or tRNA processing factors cause neurogenetic disorders in humans, such as hypomyelinating leukodystrophies and pontocerebellar hypoplasia [43]. Studies indicate that disease-causing mutations reduce Brf1 occupancy at tRNA genes to decrease Pol III gene transcription and impair cell growth [43].

**Figure 2:**
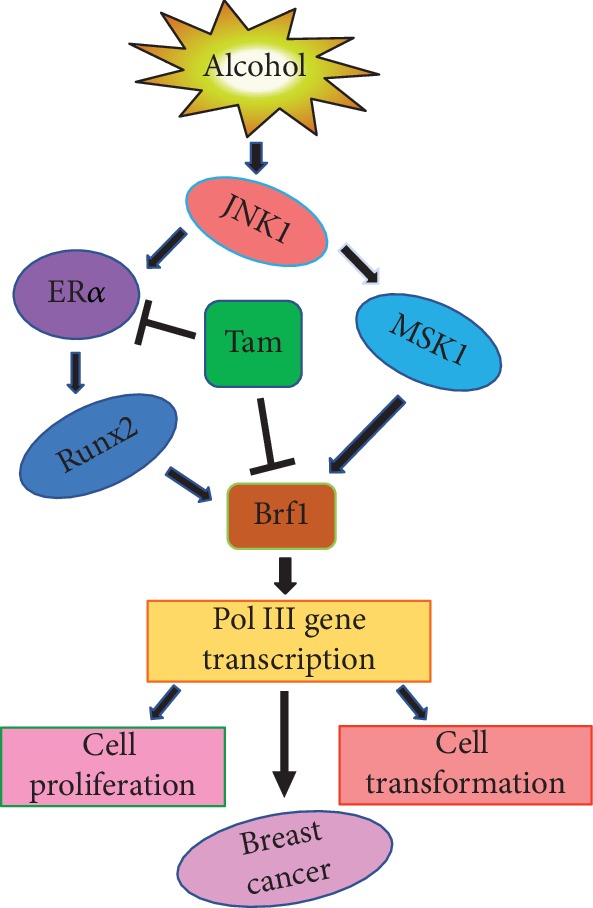
The alcohol-induced modulation of Brf1 expression. Alcohol increases Brf1 expression through two ways in breast cells. One is that alcohol activates JNK1 and MSK1 to upregulate Brf1 expression; another way is that alcohol activates JNK1 to enhance ER*α* and Runx2 expression, leading to augmenting Brf1 expression. The two pathways increase RNA Pol III gene, eventually resulting in cell proliferation and transformation and mammary tumor development, while Tam inhibits ER*α* activity and decreases the cellular level of Brf1.

Although PIK and PKB are able to phosphorylate Brf1 [40–42], our studies indicate that alcohol activates JNK1, but not JNK2 [19], while JNK1 indeed mediates alcohol-induced Brf1 expression in both hepatocellular carcinoma and breast cancer cells [19, 20]. In the ER+ breast cancer cell line, MCF-7, alcohol activates JNK1 and enhances ER*α* activity to elevate Brf1 expression [20]. The increase in Brf1 and Pol III genes by alcohol in ER+ breast cancer cell lines is significantly higher than those in ER- breast cancer cell lines and nontumor breast cell lines [20]. This finding is consistent with an early study, in which Brf1 expression in ER+ human breast cancer cases is higher than that in ER- cases [44]. Interestingly, inhibition of JNK1 signaling by its chemical inhibitor or siRNA significantly reduces ER*α* activity, resulting in the decrease in Brf1 expression [20]. In contrast, enhancing JNK1 expression augments ER*α* activity to elevate cellular levels of Brf1 [20]. Further analysis indicates that the decrease in ER*α* attenuates alcohol-caused induction of Brf1 expression [20]. Together, these studies indicate that different phosphorylation of Brf1 reveals various functions in Pol III genes. Alcohol increases ER*α* activity to enhance Brf1 expression through the JNK1 pathway (Figure 2).

### 2.5. Alcohol Increases Expression of Brf1 Causing the Alterations of Cellular Phenotypes

Studies have demonstrated that carcinogens are able to increase Brf1 expression in cell culture models. EGF (epidermal growth factor) stimulates Brf1 expression in mouse epidermal cell line to augment the rates of cell growth and cell transformation [45]. Repressing Brf1 expression inhibits the EGF-stimulated cell transformation [45], whereas DEN (diethylnitrosamine), a potent chemical hepatocarcinogen, has widely been used to induce HCC (hepatocellular carcinoma) in rodents. DEN administration caused HCC in 100% of male mice [46]. A study has indicated that DEN dramatically elevates Brf1 expression in liver cells [47]. DEN-induced cell proliferation is inhibited by decreasing the cellular level of Brf1 [47]. These studies demonstrate that carcinogens are indeed able to increase Brf1 expression [45, 47]. Alcohol has been classified as carcinogenic to humans by the IACR (International Agency for Research on Cancer) [31, 48, 49]. It implies that alcohol has potential to increase Brf1 expression, resulting in alteration of cellular phenotypes. Alcohol may be a good reagent to study the mechanism of breast cell proliferation, cell transformation, and tumor development.

Studies have demonstrated that alcohol dramatically increases Brf1 expression in ER+ breast cancer cell lines [20]. Recent studies further indicate that Brf1 is overexpressed in human HCC biopsies [17]; the high Brf1 expression of the HCC patients displays shorter survival times [17]. This indicates that the abnormal expression of Brf1 links to human cancers and high Brf1 expression predicts worse prognosis of HCC. Although alcohol significantly enhances Brf1 expression in ER+ breast cancer cells, low dose (25mM) of alcohol alone is hard to induce transformation of nontumor breast cells (MCF-10A) [20], while the MCF-10A cells treated with EGF are able to cause colony formation in soft agar assay. But, the treatment of EGF plus alcohol significantly increases the rates of cell transformation [20]. However, once Brf1 expression is repressed in MCF-10A cells, the rates are dramatically reduced in this condition. And also, the rates of cell proliferation are enhanced under alcohol treatment in both liver cells and breast cells [17, 20]. Inhibition of Brf1 expression decreases alcohol-caused rates of cell growth. These studies demonstrate that alcohol-increased Brf1 expression causes the changes in cell phenotypes.

The above studies indicate that carcinogens increase Brf1 expression *in vitro* and abnormal expression of Brf1 is associated with human cancers, such as breast cancer and HCC [10, 17]. Brf1 is overexpressed in human biopsies of liver and breast cancer cases. These evidences support the idea that abnormal expression of Brf1 is tightly linked to cell transformation and breast cancer.

### 2.6. Epigenetic Regulation of Brf1 Expression by Alcohol-Induced Histone H3 Phosphorylation

**Figure 3:**
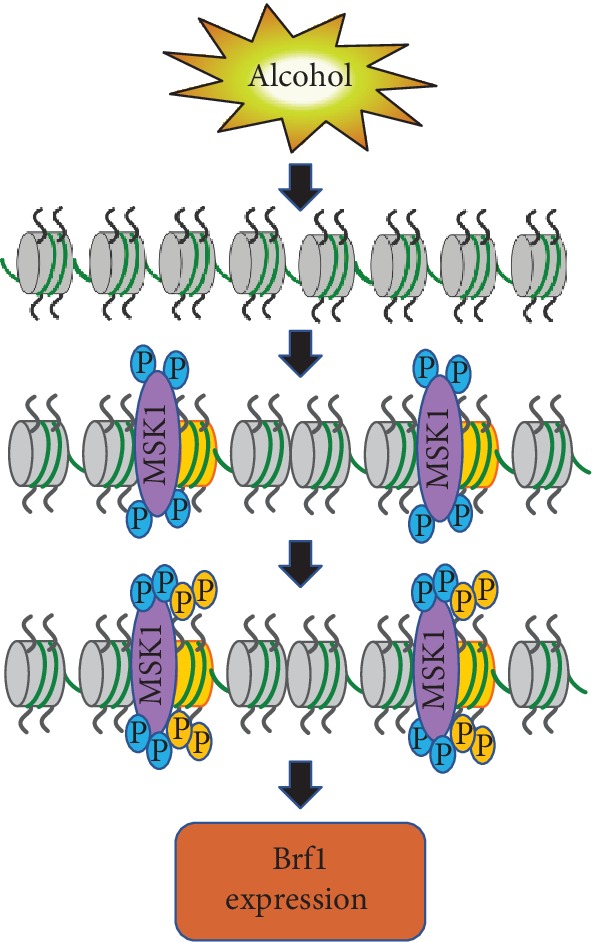
Alcohol-induced histone H3 phosphorylation which upregulates Brf1 expression. Alcohol induces activation of MSK1, which binds to the sites of chromatin of the target genes. Activated MSK1 mediates histone H3 phosphorylation (H3ph) to cause chromatin remodeling at local, leading to increases in Brf1 transcription. P: the phosphorylation group.

Gene expression is epigenetically regulated through DNA methylation and covalent chromatin modifications, such as phosphorylation (H3ph), acetylation (H3ac), and methylation (H3me) of histone H3 and other histones. Emerging evidences suggest that histone modifications, such as methylation, are a dynamic process that modulates transcriptional activity in both normal and tumor cells [50–54]. Histone modifications are the consequence of a balance of the enzymes, which involve histone modifications. Histone methylation is a result of a coordinated interplay between histone lysine methyltransferases [such as EZH2 (enhancer of zeste homolog 2)] and demethylases [such as LSD (lysine-specific demethylase) or KDM (lysine demethylase)] at specific gene promoters, thereby contributing to normal development or occurring disease [52, 55]. Triple methylation of histone H3 lysine 27 (H3K27me2/3) and lysine 9 (H3K9me3) is a hallmark of silenced chromatin [56]. In contrast, triple methylation of histone H3 lysine 4 (H3K4me3) is an activating histone mark [57, 58]. Histone H3 phosphorylation (H3ph) is mediated by protein kinase, such JNK1 and MSK1, and phosphatases [59, 60], whereas histone H3 acetylation is modulated by histone acetyltransferases and deacetylases, such as HDAC1 and HDAC2. We have reported that H3ph at serine 10 and serine 28 increases Pol III gene transcription to cause cell transformation (46.48). Inhibition of the H3ph decreases Pol III gene transcription and represses cell transformation (46.48). However, increasing H3ph occupancy in the Brf1 promoter reduces H3K27me3 binding to them, leading to the enhancement of Pol III gene transcription (46.48). Previous studies have demonstrated that MSK1 mediates H3ph [60]. Very recent studies further indicate that alcohol activates MSK1 and induces H3ph (unpublished). Inhibition of MSK1 signaling by its chemical inhibitor, SB-747651A, represses the induction of Brf1 expression caused by alcohol (unpublished). Together, the above studies show that alcohol induces Brf1 expression through the JNK1-MSK1-H3ph-ER*α* and Runx2 pathway to cause phenotypic alterations of breast cells (Figure 3).

## 3. Deregulation of RNA Pol III Genes and Breast Cancer

As mentioned above, RNA polymerase III transcribes a variety of untranslated RNAs and the Pol III gene products include tRNAs, 5S rRNAs, 7SL RNA, 7SK RNA, and U6 RNA [61–63]. Since the tRNAs and 5S rRNAs control the translational and growth capacity of cells and link to tumor development [64, 65], it is especially important to explore alcohol's role in the deregulation of Pol III genes and tumor development. An animal study has demonstrated that alcohol increases the transcription of Pol III genes [19]. The levels of tRNA and 5S rRNA transcription in the liver tumor tissues of HCV NS5A transgenic mice are much higher than that in nontumor liver tissues [19]. Alcohol feeding of mice promotes mammary tumorigenesis [36, 37]. Cell culture studies have demonstrated that the transcription products of RNA Pol III genes were elevated in both transformed and tumor cells [19, 20, 29, 33, 66–69], while the inductions of Pol III genes by alcohol in tumor cells were much higher than those in nontumor cells [33]. Consistent with this idea, enhanced Pol III gene transcription is required for oncogenic transformation of cells [19, 20, 29]. It suggests that alcohol-induced deregulation of Pol III genes play a crucial role in cell transformation and tumorigenesis [19, 20, 29, 33, 36, 37].

### 3.1. The Feature of Alcohol-Enhanced Transcription of Pol III Genes in Breast Cells

Breast cancer has become the most common cancer and leading cause of cancer mortality in women in the United States [70]. In terms of the status of ER expression, the patients of breast cancer were divided into ER+ cases and ER- cases. Approximately 80% cases of human breast cancers are ER+, and ~20% cases are ER- [2, 5, 6]. The latter include the cases of ER-, PR-, and Her-, which are called triple-negative breast cancer (TNBC). Clinical studies indicate that ER+ cases of breast cancer have good prognosis after hormone treatment, while ER- cases display worse prognosis.

As alcohol intake is associated with ER+ cases of breast cancer much more than with ER- cases of this disease [8, 9], the mechanism analysis of alcohol-associated breast cancer indicates that cellular levels of Pol III gene transcription are significantly different in ER- breast cell lines, not ER- nontumor breast cell lines (MCF-10A, MCF-10F, and MCF12A) or ER- breast cancer cell lines (MDA-MB235 and SKRB-3), from ER+ breast cancer cell lines (MCF-7 and TDT477) [20]. Interestingly, the alcohol-increased amounts of precursor tRNA^Leu^ and 5S rRNA transcript in ER+ breast cancer cells (MCF-7 and TDT477) are dramatically higher than those in ER- cells (MCF-10A, MCF12A, MDA-MB235, and SKRB-3) [10, 20, 66–69]. The induction of Pol III genes caused by alcohol in ER+ MCF-7 cells is 5-6 higher than that in ER- breast cells (MCF-10A, MCF-10F, MCF-12A, MDA-MB235, and SKRB-3). It suggests that alcohol-induced deregulation of Pol III genes is in an ER-dependent manner. The feature of alcohol-caused deregulation of Pol III genes in ER+ breast cells is consistent with that of alcohol intake which is associated with ER+ cases of breast cancer.

### 3.2. Molecular Mechanism of Alcohol-Increased RNA Pol III Gene Transcription in Breast Cancer Cells

As alcohol enhances the activity of ER*α* and activates the E2 signaling pathway in breast cells [37, 71], it suggests that ER*α* may be involved in the modulation of alcohol-induced transcription of Pol III genes. Alcohol treatment increases the activity of the ERE-Luc promoter [20, 72]. E2, a ligand of ER*α*, elevates 3-fold of the ERE-Luc activity, while alcohol plus E2 produces 4.5-fold of its activity in ER+ breast cancer cells [20]. In addition, alcohol strongly increases the cellular levels of ER*α* mRNA or protein [20]. These studies demonstrated that alcohol indeed enhances ER*α* expression in ER+ breast cancer cells. To establish whether ER*α* expression affects Pol III gene transcription, ER+ breast cancer cells (MCF-7) were treated with alcohol. The results indicate that E2 alone elevated (~2-fold) tRNA^Leu^ and 5S rRNA transcription, while alcohol plus E2 produces 11-14-fold of the increase in the transcription of Pol III genes [20]. However, inhibiting expression of ER*α* by its siRNA reduces the levels of ER*α* mRNA and protein and also decreases the transcription of tRNA^Leu^ and 5S rRNA [20]. These studies support the conclusion that ER*α* modulates alcohol-induced transcription of Pol III genes.

Tam is an estrogen receptor antagonist. Tam competitively binds to ER in tumors and other tissue targets to inhibit estrogen effects [32]. The traditional concept of Tam efficacy in breast cancer therapy is known through the ER*α* pathway. Recent studies demonstrate that Tam is able to directly inhibit alcohol-induced transcription of Brf1 and Pol III genes [33]. This function of Tam may explain its efficacy in ER+ cases of breast cancer, but not TNBC cases. This new finding provides the possibility that inhibition of Pol III gene transcription may be a potential approach to repress alcohol-promoted cell transformation and breast cancer development and to elevate the efficacy in Tam-resistant cases of ER+ breast cancer.

In addition, very recent studies indicate that Runx2 is overexpressed in human biopsies of breast cancer (unpublished). Alcohol enhances Runx2 expression in ER+ breast cancer cells. Repression of Runx2 decreases Brf1 expression and Pol III gene transcription. Further analysis indicates that Runx2 and Brf1 colocalize in the nucleus and they synergistically modulate Pol III gene transcription. Because ER*α* mediates alcohol-induced Runx2 expression in ER+ MCF-7 cells, the above studies demonstrate that Runx2 modulates Brf1 expression and Pol III gene transcription through the ER*α* pathway induced by alcohol.

### 3.3. Brf1 Plays a Critical Role in Alcohol-Induced Deregulation of Pol III Genes

As Brf1 is a key transcription factor to directly regulate Pol III gene activity, once the levels of Brf1 are changed in any physiological and pathological conditions, the cellular levels of tRNAs and 5S rRNAs would be altered, resulting in changes in cellular phenotype or occurrence of diseases, even tumor development. As mentioned above, these factors, such as signaling molecules (such as JNK1 and MSK1), hormone receptor (such as ER*α*), transcription factors (such as Runx2, BRCA1), epigenetic modifications (such as histone H3 phosphorylation), carcinogens (such as alcohol, EGF, DEN), medicine (Tam), and others [ [20, 31, 33, 66–69, 73] and (unpublished)], which impact Brf1 expression, lead to deregulation of Pol III genes and cause cell proliferation, cell transformation, and eventually breast cancer development. The alcohol-induced deregulation of RNA Pol III genes is a consequence of Brf1 expression change in ER+ breast cells. In terms of the feature of Brf1 in breast tissue, it strongly implies that developing an inhibitor to repress Brf1 expression may reach to the therapeutic purpose of human breast cancer.

## 4. Summary and Prospects

Alcohol is a liquid diet and widely used as consumption drink in daily life. Alcohol has been classed as a carcinogen to human by IACR in 2011 [31, 48, 49]. Alcohol intake-associated diseases have caused more and more attention. It has especially shown solicitude for that alcohol consumption is consistent with the risk of breast cancer in women. Studies have demonstrated that Brf1 is overexpressed in ER+ cases of breast cancer. High expression of Brf1 in ER+ cases of this disease shows better prognosis under the hormone treatment. Further studies indicate that a hormone drug, Tam, is able to directly repress Brf1 expression, causing the reduction of Pol III gene transcription except Tam competitively binding to ER. Mechanism studies have demonstrated that ER*α* affects the TFIIIB subunit and Brf1 expression, but not TBP, in breast cells. This feature of Brf1 in breast cancer provides a possibility to develop a new approach by inhibiting Brf1 expression for therapy of the patients of breast cancer. Brf1 is not only a therapeutic target of breast cancer but also a biomarker of prognosis of the disease. In the future, more and more attention should be immersed into the studies of the Brf1 inhibitor as a novel approach of breast cancer therapy. It will be of huge interest for both basic science and clinical communities to investigate this mechanism of alcohol-associated breast cancer and identify whether reducing Brf1 expression and Pol III gene transcription by direct or indirect manners represses mammary tumor development.

## Abbreviations

Brf1: TFIIB-related factor 1
TBP: Tata-box binding protein
Pol III gene: RNA polymerase III-dependent gene
IARC: International Agency for Research on Cancer
PNCs: Perinucleolar compartments
RNA Pol: RNA polymerase
OS: Overall survival
DFS: Disease-free survival
TNBC: Triple-negative breast cancer
Tam: Tamoxifen
ALD: Alcoholic liver disease
ROS: Reactive oxygen species.
